# Oral secretory leukocyte protease inhibitor (SLPI): Associations with oropharyngeal cancer and treatment outcome

**DOI:** 10.1371/journal.pone.0254161

**Published:** 2021-07-02

**Authors:** Brittney L. Dickey, Bradley Sirak, Laura Martin-Gomez, Richard R. Reich, Martha Abrahamsen, Kimberly Isaacs-Soriano, Christine H. Chung, Anna R. Giuliano

**Affiliations:** 1 Center for Immunization and Infection Research in Cancer, H. Lee Moffitt Cancer Center and Research Institute, Tampa, Florida, United States of America; 2 Department of Cancer Epidemiology, H. Lee Moffitt Cancer Center and Research Institute, Tampa, Florida, United States of America; 3 Biostatistics and Bioinformatics Shared Resource, H. Lee Moffitt Cancer and Research Institute, Tampa, Florida, United States of America; 4 Department of Head and Neck-Endocrine Oncology, H. Lee Moffitt Cancer and Research Institute, Tampa, Florida, United States of America; University of Wisconsin, UNITED STATES

## Abstract

**Background:**

Rates of oropharyngeal cancer (OPC) associated with alcohol & tobacco use have decreased, while human papillomavirus (HPV) associated OPC has increased among men in the US. Secretory leukocyte protease inhibitor (SLPI), detectable in a variety of secretions, has been implicated in cancers of the head and neck, associated with tumor progression and anti-viral activity. Using the recently verified oral gargle specimen, this study aimed to assess the association of salivary SLPI expression with risk of OPC and response to treatment.

**Methods:**

A case-control study design compared levels of salivary SLPI among OPC cases to age and tobacco smoking matched healthy controls. Oral HPV DNA and SLPI was quantified from oral gargle specimens. Logistic regression estimated odds ratios (OR) and 95% confidence intervals (CI) for associations of oral SLPI and risk of OPC and treatment outcomes.

**Results:**

In crude and adjusted analyses of 96 OPC cases and 97 age- and smoking-matched controls, OPC was not significantly associated with oral gargle SLPI levels. Among cases, oral SLPI was associated with tonsillectomy (p = 0.018) and among controls oral SLPI was associated with HPV in the oral gargle (p = 0.008). Higher concentrations of SLPI was significantly associated with increased odds of incomplete treatment response (T2: OR: 12.39; 95% CI: 1.44–106.72; T3: OR: 9.86; 95% CI: 1.13–85.90) among all cases, but not among P16+ cases.

**Conclusions:**

Salivary SLPI was not associated with OPC risk but was associated with higher odds of an incomplete treatment response.

## Introduction

Head and neck cancers (HNC), which include tumors of the oral cavity, oropharynx, larynx, and hypopharynx, are associated with lifestyle factors including tobacco and alcohol use. One sub-site, the oropharynx, is also highly associated with human papillomavirus (HPV) infection [1, 2]. Over the last decade, rates of oropharyngeal cancer (OPC) associated with tobacco and alcohol use have decreased, while HPV-associated OPC has increased among men particularly in the US and other high resource settings [2, 3]. Tumor progression and outcomes also differ by OPC etiologies with metastases higher, but response to treatment and survival better among HPV-associated OPC compared to tobacco/alcohol-associated OPC [4–6]. The immune environment associated with the development of a virally caused OPC remains under investigation.

One immune factor, secretory leukocyte protease inhibitor (SLPI), has been implicated in HNC as a possible marker of tumor progression [7]. Higher levels of SLPI in the tumor have been associated with reduced metastases in HNC. In one such study, SLPI expression was 5.9-fold lower in primary metastatic HNC cases compared to non-metastatic cases of HNC [8]. As a protease inhibitor, it has been suggested that SLPI provides protection to the mucosa and skin against the enzymatic pathways that lead to cancer invasion and progression by preventing tissue degradation from certain tumor proteases [9, 10].

Regarding OPC, SLPI has also been shown to have anti-viral properties; with prior studies suggesting SLPI blocks the binding of HIV and HPV to host cells [10–12]. In a sample of 54 HNC cases, those with absent/weak tumor SLPI expression were more likely to be HPV positive [13]. There is, however, scarce data to conclude any temporal relationship between SLPI and OPC incidence or tumor progression, based on HPV-status. This is further complicated by the fact that only 2% of men aged 18–73 may be infected with an oral, oncogenic HPV infection and only 0.6% with an oral, oncogenic HPV16 infection, the genotype most responsible for HPV-associated OPC [14].

Until recently, SLPI had only been quantified in HNC tumor specimens. However, SLPI can be detected in a variety of secretions, with high concentrations found in saliva [10]. More recently it was quantified in oral gargle specimens, a useful specimen for cancer screening that has been routinely utilized to also assess oral HPV status [15]. While concordance between tumor and oral gargle SLPI is unknown, we have previously found high concordance of other biomarker measures between tumor and oral gargle specimens in men diagnosed with OPC [16].

Therefore, using the oral gargle specimen as a surrogate specimen, we conducted a case-control study to evaluate the association of oral salivary SLPI expression with risk of OPC, and further evaluated response to treatment among OPC cases. We hypothesized that, like prior studies of HNC, higher levels of SLPI would be protective for risk of OPC and lead to better treatment outcomes.

## Materials and methods

### Study population

A case-control study design was used to compare oral levels of SLPI among OPC cases to age and smoking frequency matched controls. Cases were selected from an ongoing study of OPC biomarkers, which recruited men with OPC from the Head and Neck Oncology and Radiation Oncology clinics at the Moffitt Cancer Center from May 2014 to March 2017. Approval was obtained from Advarra Institutional Review Board and the Moffitt Cancer Center Scientific Review Committee. Briefly, patients were pre-screened for eligibility via medical records and eligible men were approached by a trained clinical coordinator for study participation. Interested and eligible men signed informed consent. To ensure generalizability to the larger population of men diagnosed with OPC who may be candidates for biomarker evaluation, eligible cases included men 18 years and older, with a histologically confirmed, and newly diagnosed squamous cell carcinoma of the oropharynx (n = 113). Participants were excluded if they had recurrent OPC, received treatment prior to enrollment or did not complete the study survey (n = 17).

The HPV Infection in Men (HIM) Study was used to identify OPC-free controls. Briefly, the HIM study included men from the United States, Brazil, and Mexico who were recruited from March 2005 to September 2009 through media advertising, educational presentations, or during routine urogenital or sexual health care. Eligible subjects were followed every 6 months and included men (1) age of 18–70 years; (2) residing at one of the study sites; (3) with no previous diagnosis of penile or anal cancers; (4) with no previous diagnosis of genital warts; (5) with no symptoms of a sexually transmitted infection (STI) and no current receipt of STI treatment; (6) with no current participation in an HPV vaccine study; (7) with no history of human immunodeficiency virus (HIV) infection or AIDS; (8) with no history of imprisonment, homelessness, or drug-abuse treatment during the past 6 months; and (9) willing to comply with 10 scheduled visits every 6 months for 4 years and no plans to relocate during that time. Complete, detailed description of the HIM study methods has been previously published [17, 18]. Approval was obtained from the human subjects committees of the University of South Florida (United States), Ludwig Institute for Cancer Research (Brazil), Centro de Referencia e Treinamento em Doencas Sexualmente Transmissíveis e AIDS (Brazil), and Instituto Nacional de Salud Publica de Mexico (Mexico). All participants gave written informed consent. For this study controls were selected from the men recruited in Tampa, Florida (USA) (same location as cases) that also had an archived oral gargle supernatant and completed questionnaire data available for study (n = 527). Participants from the Tampa cohort of the HIM study were recruited from the greater Tampa metropolitan area or University of South Florida via flyers, posters, mail, and media announcements from June 2005 to September 2009 and followed every 6 months through 2016. They were matched to cases on age and smoking status (current, former, or never smoker) (n = 97).

### Data collection

In both studies, demographics, medical history, smoking and alcohol use, sexual activity, and oral health history were obtained using a self-administered questionnaire. All participants also provided an oral gargle sample. Briefly, participants were asked to swish-gargle with 15mL of mouthwash (Up&Up®) for 30 seconds before returning the sample to the collection vial. Among OPC cases, electronic medical records were reviewed for response to treatment at three, six, twelve, and eighteen-month post treatment clinical time points.

### Laboratory analyses

#### HPV extraction

Oral HPV DNA was extracted from oral gargle cell pellets using the automated BioRobot MDx (Qiagen). HPV status of oral gargle specimens for all participants was obtained using the HPV SPF_10_ PCR-DEIA-LiPA_25_ line probe assay (DDL Diagnostic Laboratory, Rijswik, the Netherlands) as previously published [19]. For cases, immunostaining for p16^INK4a^ (p16), a surrogate marker of HPV presence in the tumor, was completed on available FFPE samples and reviewed by qualified pathologists.

#### SLPI measurement

Analysis for SLPI protein expression was completed using the Human SLPI Quantikine ELISA Kit (DP 100, R&D Systems, Minneapolis, MN, US). Oral gargle supernatants were diluted 1:200 and duplicate specimens and standards (100μl each) were assayed. A standard curve was created for each run and the optical density of the samples was used to estimate salivary SLPI concentrations in the oral gargles (ng/mL). Measurements for each individual sample in the set of duplicates were averaged to obtain a single value per specimen.

### Statistical analysis

The non-parametric Fisher’s exact test was used to compare characteristics between cases and controls; and associations with SLPI tertile by case-control status. For the case-control comparison median SLPI concentrations (ng/ml) and inter-tertile ranges (ITR) were calculated based on the distribution among controls and a categorical variable was created using ITR as follows; T1 (< 317.81), T2 (317.82–696.84) and T3 (> 696.85). Univariate and multivariate logistic regression was used to estimate odds ratios (OR) and 95% confidence intervals (CI) for associations between oral SLPI with the risk of OPC among cases and controls. Factors included in the final multivariable model were those associated with case status in Table 1 and also associated with SLPI (Table 2).

**Table 1:** Socio-demographic characteristics of OPSCC cases and disease-free controls.

Characteristics	Cases (n = 96)		Controls (n = 97)		p-value*
	N	%	N	%	
**Race**					
White	90	93.75	74	76.29	**0.002**
Black	4	4.17	17	17.53	
Other^a^	2	2.08	6	6.19	
**Ethnicity**					
Hispanic	3	3.13	11	11.34	**0.049**
Non-Hispanic	93	96.88	86	88.66	
**Age at baseline (years)**					
Median (range)	60 (37–79)		61 (35–83)		0.880
35–49	11	11.46	12	12.37	
50–59	30	31.25	33	34.02	
60–69	25	36.46	30	30.93	
≥70	20	20.83	22	22.68	
**Marital status**					
Married/cohabiting	64	66.67	61	62.89	0.546
Single/divorced/separated/widowed	31	32.29	36	37.11	
Refused or N/A	1	1.04	0	0	
**Education**					
High school (<12 years)	26	27.08	12	12.37	**0.041**
Some college/vocational school	28	29.17	35	36.08	
College graduate	22	22.92	33	34.02	
Postgraduate/professional school	19	19.79	17	17.53	
Refused or N/A	1	1.04	0	0	
**Smoking**					
Current	8	8.33	6	6.19	0.772
Former	47	48.96	46	47.42	
Never	41	42.71	45	46.39	
**Cigarette (pack-years)**					
Never	41	42.71	45	46.39	**0.007**
≤5	9	9.38	23	23.71	
6–29	20	20.83	18	18.56	
≥30	25	26.04	10	10.31	
N/A or refused	1	1.04	1	1.03	
**Alcohol drinks per occasion in the past month**					
None	38	39.58	29	29.9	0.291
1–4	51	53.13	57	58.76	
≥5	7	7.29	11	11.34	
**Lifetime number of people kissed with tongue**					
None	4	4.17	7	7.22	0.444
1–9	28	29.17	31	31.96	
10–24	25	26.04	22	22.68	
25–49	15	15.63	20	20.62	
≥50	22	22.92	13	13.4	
N/A or refused	2	2.08	4	4.12	
**Gave oral sex in the past 6 months**					
Yes	36	37.5	45	46.39	0.405
No	55	57.29	46	47.42	
N/A or refused	5	5.21	6	6.13	
**Tonsillectomy**					
Yes	37	38.54	12	13.37	**<0.001**
No	57	59.38	85	87.63	
N/A or refused	2	2.08	0	0	
**Time since tonsillectomy (years ago)**					
<2 years	6	6.26	0	0	**<0.001**
2–29 years	1	1.04	0	0	
30+ years	28	29.17	12	12.37	
N/A or Refused	4	4.17	0	0	
**Gingivitis**					
No	75	78.13	71	73.2	0.747
Yes	20	20.83	25	25.77	
N/A or refused	1	1.04	1	1.03	
**Teeth extracted prior to diagnosis**					
0	42	43.75	58	59.79	**0.033**
<10	31	32.29	30	30.93	
≥10	18	18.75	7	7.22	
N/A or refused	5	5.21	2	2.06	
**Tumor location**					
Tonsil	47	48.96	-		-
BOT	45	46.88			
Other OP	4	4.17			
**Tumor Stage (AJCC 7**^**th**^ **edition)**					
Stages I, II, and III	25	26.04	-		-
Stages IV (A, Band C)	71	73.96			
**p16**^**INK4**^^**a**^ **(IHC)**					
Positive	77	80.21	-		-
Negative	11	11.46			
N/A	8	8.33			
**HPV status in oral gargle**					
Positive	88	92.63	28	32.18	**<0.001**
Negative	7	7.37	59	67.82	
**HPV status in tumor**					
Positive	70	93.33	-		-
Negative	5	6.67			

* The non-parametric p-value is calculated using Fisher’s exact test for a significance of *p<0*.*05*.

^a^ Other race includes American Indian, Alaska Native, Native Hawaiian, Asian/Pacific Islander, and mixed race.

**Table 2:** Associations of oral SLPI with patient characteristics among cases and controls.

	SLPI (Cases)				SLPI (Controls)			
	T1	T2	T3	p-value	T1	T2	T3	p-value
	N (%)	N (%)	N (%)		N (%)	N (%)	N (%)	
**Race**								
White	26 (86.7)	26 (100)	38 (95.0)	0.319	22 (66.7)	26 (81.2)	26 (81.3)	0.194
Black	3 (10.0)	0 (0)	1 (2.5)		8 (24.2)	3 (9.4)	6 (18.7)	
Other^a^	1 (3.3)	0 (0)	1 (2.5)		3 (9.1)	3 (9.4)	0 (0)	
**Ethnicity**								
Hispanic	2 (6.7)	0 (0)	1 (2.5)	0.476	7 (21.2)	3 (9.4)	1 (3.1)	0.074
Non-Hispanic	28 (93.3)	26 (100)	39 (97.5)		26 (78.8)	29 (90.6)	31 (96.9)	
**Age at baseline (years)**								
18–49	4 (13.3	4 (15.4)	3 (7.5)	0.677	6 (18.2)	2. (6.2)	4 (12.5)	0.458
50–59	7 (23.3)	8 (30.8)	15 (37.5)		13 (39.4)	11 (34.4)	9 (28.1)	
60–69	14 (46.7)	9 (34.6)	12 (3.0)		6 (18.2)	11 (34.4)	13 (40.6)	
70+	5 (16.7)	5 (19.9)	10 (25.0)		8 (24.2)	8 (25.0)	6 (18.8)	
**Marital Status**								
Single, divorced, widowed	10 (33.3)	9 (34.6)	12 (30.0)	0.708	13 (39.4)	11 (34.4)	12 (37.5)	0.964
Married or cohabiting	20 (66.6)	16 (61.5)	28 (70.0)		20 (60.6)	21 (65.6)	20 (62.5)	
NA or refused	0 (0)	1 (3.8)	0 (0)		0 (0)	0 (0)	0 (0)	
**Education**								
≤12/ general education	9 (30)	7 (26.9)	10 (25.0)	0.552	6 (18.2)	3 (9.4)	3 (9.4)	0.416
Some college	8 (26.7)	6 (23.1)	14 (35.0)		12 (36.4)	11 (34.4)	12 (37.5)	
College graduate	7 (23.3)	4 (15.4)	11 (27.5)		13 (39.4)	11 (34.4)	9 (28.1)	
Postgraduate-professional	6 (20.0)	8 (30.8)	5 (12.5)		2 (6.0)	7 (21.8)	8 (25.0)	
NA or refused	0 (0)	1 (3.8)	0 (0)		0 (0)	0 (0)	0 (0)	
**Cigarette (pack-years)**								
Never	15 (50.0)	9 (34.6)	17 (42.5)	0.841	17 (51.5)	15 (46.9)	13 (40.6)	0.900
≤5	3 (10.0)	3 (11.5)	3 (7.5)		9 (27.3)	6 (18.8)	8 (25.0)	
6–29	4 (13.3)	7 (26.9)	9 (22.5)		5 (15.2)	6 (18.8)	7 (21.9)	
30+	7 (23.3)	7 (26.9)	11 (27.5)		2 (6.1)	4 (12.5)	4 (12.5)	
NA or refused	1 (3.3)	0 (0)	0 (0)		0 (0)	1 (3.1)	0 (0)	
**Alcohol drinks per occasions in the past month**								
No alcohol	10 (33.3)	12 (46.2)	16 (40.0)	0.649	11 (33.3)	10 (31.3)	8 (25.0)	0.510
1–4 drinks	16 (53.3)	13 (50.0)	22 (55.0)		20 (60.6)	16 (50.0)	21 (65.6)	
5+ drinks	4 (13.3)	1 (3.8)	2 (5.0)		2 (6.1)	6 (18.7)	3 (9.4)	
**Lifetime number of people kissing with tongue**								
0	2 (6.7)	2 (7.7)	0 (0)	0.100	2. (6.1)	4 (12.5)	1 (3.1)	0.796
1–9	5 (16.7)	6 (23.1)	17 (42.5)		9 (27.3)	9 (28.1)	13 (40.6)	
10–24	10 (33.3)	9 (34.6)	6 (15.0)		10 (30.3)	5 (15.6)	7 (21.9)	
25–49	4 (13.4)	3 (11.5)	8 (20.0)		5 (15.1)	8 (25.0)	7 (21.9)	
≥50	7 (23.3)	6 (23.1)	9 (22.5)		5 (15.1)	5 (15.6)	3 (9.4)	
NA or refused	2 (6.7)	0 (0)	0 (0)		2 (6.1)	1 (3.1)	1 (3.1)	
**Gave oral sex in the past 6 months**								
Yes	8 (26.7)	10 (38.4)	18 (45.0)	0.442	16 (48.5)	15 (46.9)	14 (43.8)	0.121
No	19 (63.3)	15 (57.7)	21 (52.5)		12 (36.4)	17 (53.1)	17 (53.1)	
NA or refused	3 (10.0)	1 (3.9)	1 (2.5)		5 (15.1)	0 (0)	1 (3.1)	
**Tonsillectomy**								
Yes	5 (16.6)	13 (50.0)	19 (47.5)	**0.018**	4 (12.1)	3 (9.4)	5 (15.6)	0.807
No	24 (80.0)	13 (50.0)	20 (50.0)		29 (87.9)	29 (90.6)	27 (84.4)	
NA or refused	1 (3.3)	0 (0)	1 (2.5)		0 (0)	0 (0)	0 (0)	
**Time since tonsillectomy (years)**								
Never	24 (80.0)	13 (50.0)	20 (50.0)	0.073	29 (87.9)	29 (90.6)	27 (84.4)	0.807
≤2	0 (0)	1 (3.9)	5 (12.5)		0 (0)	0 (0)	0 (0)	
2–29	0 (0)	1 (3.9)	0 (0)		0 (0)	0 (0)	0 (0)	
30+	5 (16.7)	10 (38.4)	13 (32.5)		4 (12.1)	3 (9.4)	5 (15.6)	
NA or refused	1 (3.3)	1 (3.9)	2 (5.0)		0 (0)	0 (0)	0 (0)	
**Gingivitis**								
Yes	8 (26.7)	3 (11.5)	9 (22.5)	0.314	8 (24.3)	9 (28.1)	8 (25.0)	1.000
No	21 (70.0)	23 (88.5)	31 (77.5)		24 (72.7)	23 (71.9)	24 (75.0)	
NA or refused	1 (3.3)	0 (0)	0 (0)		1 (3.0)	0 (0)	0 (0)	
**Teeth extracted prior to diagnosis**								
0	9 (30.0)	14 (53.9)	19 (47.5)	0.401	21 (63.6)	18 (56.2)	19 (59.4)	0.640
<10	13 (43.3)	7 (26.9)	11 (27.5)		9 (27.3)	11 (34.4)	10 (31.2)	
≥10	5 (16.7)	5 (19.2)	8 (20.0)		1 (3.0)	3 (9.4)	3 (9.4)	
NA or refused	3 (10.0)	0 (0)	2 (5.0)		2 (6.1)	0 (0)	0 (0)	
**HPV status in the oral gargle**								
Positive	26 (86.7)	25 (100)	37 (92.5)	0.167	13 (46.4)	11 (40.7)	4 (12.5)	**0.008**
Negative	4 (13.3)	0 (0)	3 (7.5)		15 (53.6)	16 (59.3)	28 (87.5)	
HPV Status in the tumor								
Positive	19 (95.0)	21 (87.5)	30 (96.8)	0.509	--	--	--	--
Negative	1 (5.0)	3 (12.5)	1 (3.2)		--	--	--	
Tumor Stage 4								
Yes	22 (73.3)	19 (73.1)	30 (75.0)	1.000	--	--	--	--
No	8 (26.7)	7 (26.9)	10 (25.0)		--	--	--	

* The non-parametric p-value is calculated using Fisher’s exact test for a significance of *p<0*.*05*.

SLPI tertiles: T1: < 317.81 ng/mL; T2: 317.82–696.84 ng/mL; and T3: >696.85 ng/mL.

^a^ Other race includes American Indian, Alaska Native, Native Hawaiian, Asian/Pacific Islander, and mixed race.

To investigate treatment outcomes among cases, SLPI tertiles were created using median SLPI and ITR among cases. Case-based SLPI tertiles were as follows: T1 (< 342.64), T2 (342.65–744.42) and T3 (>744.43). Cases were grouped as having a complete response or incomplete response to therapy at three months. Complete response was the inability to detect tumor in imaging (CT or PET-CT scan) and by physical response. Incomplete response included partial response, or tumor shrinkage with respect to pre-treatment, or progressive disease. Seven patients were excluded because they were lost to follow-up, did not complete treatment or were treated elsewhere. Univariate logistic regression was used to assess the association with oral SLPI concentration and OPC treatment outcomes at three months post-treatment evaluation among all cases and, because prognosis is better for HPV-positive cases, among those positive for P16 only. Due to small sample size, adjustment for confounders was not plausible and therefore, only a crude odds ratio was reported. Finally, progression-free survival for the 36 months following therapy initiation stratified by SLPI tertile was assessed using a Kaplan-Meier curve with log-rank test hazard ratios (HR) among all cases and P16+ cases only. For all analyses statistical significance was defined as alpha of 0.05. All statistical analyses were completed using SAS version 9.4 (SAS Institute, Cary, NC, US).

## Results

Included on study were 96 eligible OPC cases and 97 age- and smoking-matched controls identified from the HIM study. The groups differed significantly by race (p = 0.002), ethnicity (p = 0.049) and education status (p = 0.041). Cases had significantly more (> = 30) pack-years of cigarettes smoked (p = 0.007), were more likely to have had a tonsillectomy (p<0.001) and more (> = 10) teeth extracted (p = 0.033). Cases were also significantly more likely to be oral HPV-positive (p = <0.001) with 68% HPV-positive compared to only 2% of controls (Table 1).

Oral SLPI was associated with tonsillectomy among cases only (p = 0.018) and was associated with oral HPV status (p = 0.008) among controls only. Cases with a history of tonsillectomy were more likely to have a SLPI concentration in the highest tertile (47.5%), compared to those without who were more likely to have a SLPI concentration in the lowest tertile (80.0%). HPV-positive controls were least likely to have a SLPI concentration in the highest tertile (12.5%), while HPV-negative controls were more likely to have a high SLPI concentration (87.5%). Among cases, HPV status in the oral gargle was not significantly associated SLPI tertile, though few cases were HPV-negative (Table 2).

In crude and adjusted analyses, OPC was not significantly associated with oral gargle SLPI levels (Table 3).

**Table 3:** Association between oral SLPI with risk of OPC.

SLPI tertile^a^	Cases (n)	Controls (n)	Crude OR (95%CI)	aOR ^b^ (95%CI)
**1**	30	33	REF	REF
**2**	26	32	0.89 (0.44–1.83)	0.59 (0.22–1.56)
**3**	40	32	1.38 (0.70–2.71)	1.77 (0.66–4.80)

^**a**^ SLPI tertiles: T1: < 317.81 ng/mL; T2: 317.82–696.84 ng/mL; and T3: >696.85 ng/mL.

^b^ Adjusted Odds Ratio: conditional logistic regression model adjusting for the following factors: previous **tonsillectomy** and **HPV status in the oral gargle**.

When investigating OPC treatment outcomes (Table 4), those with higher concentrations of SLPI had significantly higher odds of having an incomplete treatment response in univariate analyses only (T2: OR: 12.39; 95% CI: 1.44–106.72; T3: OR: 9.86; 95% CI: 1.13–85.90). When limited to cases associated with HPV (P16+), this association was no longer significant, though most cases that were not P16+ fell into the lowest SLPI tertile.

**Table 4:** Association of oral SLPI with risk of incomplete response to therapy at 3 months post-treatment evaluation among OPC cases and P16+ OPC cases.

SLPI tertile^a^	All Cases (n = 89)^b^			P16+ Cases (n = 72)^b^		
	Incomplete^c^ response (n)	Complete^d^ response (n)	OR (95%CI)	Incomplete^c^ response (n)	Complete^d^ response (n)	OR (95%CI)
**1**	1	31	REF	1	21	REF
**2**	8	20	**12.39 (1.44–106.72)**	7	19	7.74 (0.87–68.80)
**3**	7	22	**9.86 (1.13–85.90)**	6	18	7.00 (0.77–63.72)

^a^ SLPI tertiles: T1: < 342.64 ng/mL; T2: 342.65–744.42 ng/mL; and T3: >744.43 ng/mL.

^b^ Among all cases n = 7 were not included because they were lost to follow-up, did not complete treatment or were treated elsewhere. In addition to this, among P16+ cases, some (n = 8) did not have available tumor tissue for P16 testing.

^c^ Incomplete responses is a partial response or progressive disease.

^d^Complete responses is defined no clinical signs of disease.

**Fig 1:**
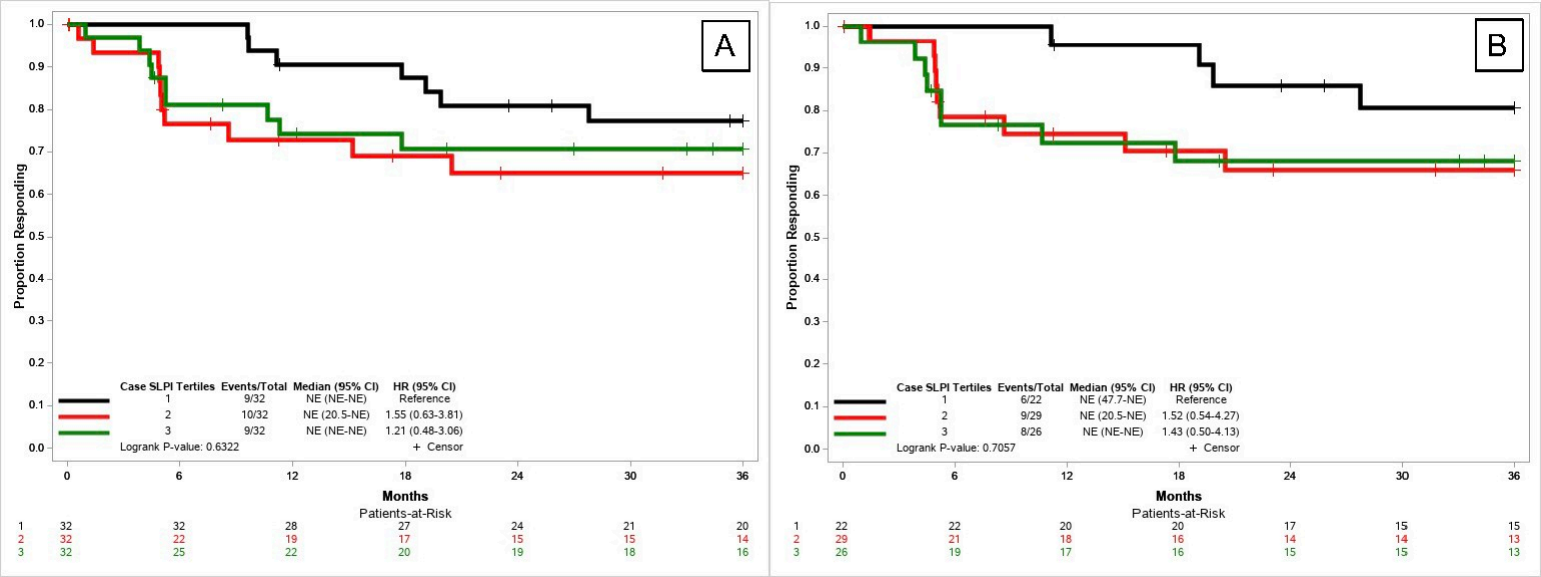
Progression free survival (progressive disease or death) of A) all cases and B) P16+ cases by oral SLPI tertile. SLPI tertiles include T1: < 342.64 ng/mL; T2: 342.65–744.42 ng/mL; and T3: >744.43 ng/mL.

While not statistically significant, among all cases in SLPI tertiles 2 and 3 there was an elevated risk of progressive disease or death in the first 36 months post-treatment (T2: HR: 1.55; 95% CI: 0.68–3.81; T3: HR: 1.21; 95% CI: 0.48–3.06) compared to the lowest SLPI tertile (Fig 1). This was also observed among P16+ cases.

## Discussion

This study investigated whether higher oral SLPI was associated with OPC and OPC treatment outcomes. Overall, higher concentrations of oral SLPI did not increase odds of OPC. Among OPC cases, higher concentrations of oral SLPI was significantly associated with an incomplete response at 3 months post-treatment evaluation but was not significantly associated with progression-free survival through three years of follow-up.

In contrast to the results of the current study, prior studies found higher levels of SLPI to be associated with reduced cancer risk and metastases. Specifically, *in vitro*, SLPI was inversely associated with tumor progression and invasion of oral squamous cell carcinoma [7]; and in tumor specimens, gene and protein levels of SLPI were lower in metastatic HNC compared to non-metastatic HNC [8]. Our study of extracellular salivary SLPI found odds of OPC to be higher, albeit non-significantly, with increasing levels of salivary SLPI. A major difference between studies is measurement of SLPI from an oral gargle specimen compared to the tumor specimen. As such, it remains unclear whether SLPI is associated with OPC and which biological specimen is most meaningful when studying this association.

Salivary SLPI is associated with viral activity and appears to possess anti-viral capabilities against HIV and HPV. Herpes Simplex Virus (HSV), for example, downregulates SLPI as an immune evasion strategy, and a potential explanation of increased risk of HIV following HSV infection [20]. HIV infection itself is inhibited in the oral cavity by higher concentrations of salivary SLPI compared to other tissues [10]. Finally, a recent study found HPV 16 cell entry was blocked when SLPI interacted with the host binding site [12]. In the current study among the controls, there was some indication that HPV infection in the oral gargle was more likely to occur at lower SLPI concentrations. However, the relationship was not the same among cases and due to the small sample size of HPV negative cases we were unable to explore this association further. A similar relationship with HPV was also observed in a prior study of HNC cases in which lower tumor SLPI levels were associated with increased tumor burden, or node status, but was found only in the absence of an HPV infection [13]. The authors also found no significant correlation between intratumoral SLPI and smoking among HNC cases. However, they did note a significant inverse correlation between smoking and HPV status, suggesting increased SLPI in response to smoking may reduce susceptibility to HPV infection. The same relationship between smoking and HPV infection was also noted in a separate study of 307 HNC cases [21]. Interestingly, that study also examined the effect of SLPI protein expression, HPV status, and smoking on progression-free survival. Patients with lower SLPI expression showed better overall survival compared to those with moderate/strong SLPI expression. Our results are similar in that cases with lower salivary SLPI concentration had better survival. Similarly, we found that higher SLPI concentrations in the oral gargle was significantly associated with increased odds of incomplete response to therapy at three months.

This study has limitations that should be noted. First, the small sample size did not allow for stratified analyses by HPV status and limited the number of variables that could be adjusted in each model. By matching on age and smoking status, two key confounders were accounted for in the study design alone, though it should be noted that while we successfully achieved matching on smoking history as defined as current, former, or never smoker, total pack-years differed significantly between groups. Future studies with larger sample size and a larger number of HPV-negative cases are needed to assess associations stratified by HPV status.

Second, this study utilized oral gargle specimens to measure salivary SLPI concentration which differed from other studies investigating cancers of the head and neck, making comparison between this and prior studies difficult. In head and neck tumor tissues, for example, lower SLPI concentration was associated with metastatic disease (8), while this study observed higher SLPI concentrations among OPC cases. It is not known how tumor tissue SLPI correlates to salivary SLPI and could present different measurements and cancer prediction probabilities. While this study did not assess tumor SLPI, salivary SLPI was measured using previously optimized and published methods [15] and may be an improved method for measuring the association between salivary HPV and SLPI concentrations. Future studies should aim to compare SLPI concentration concurrently in the tumor tissue and saliva of patients to determine the association between the two and how it relates to OPC. Finally, we previously showed that salivary SLPI declines with age, consistent with the understanding that immune function declines with age. The median age of this study population was 60 years; lack of association between SLPI and OPC and treatment outcomes may be due to overall diminished SLPI concentrations in this population. However, age-matching likely limits any overall confounding effect age may have had to the SLPI concentrations of this study population. Finally, it should be noted that treatment and its associated outcomes differs by HPV presence in the tumor, with HPV-associated tumors often responding better to treatment and surviving longer compared to non-HPV associated tumors. At the time of study we did not have treatment type, but have presented SLPI concentration by stage and HPV status, as well as treatment outcomes and survival for HPV-associated cases only.

The findings of this study are the first steps in understanding associations between salivary SLPI and risk of OPC in men only. To improve overall generalizability, future studies should include assessment of SLPI in a larger sample size with more HPV positive controls. While OPC is increasing among men in the U.S., women are also diagnosed with OPC and should be included in future studies.

In conclusion, salivary SLPI was not associated with OPC but was associated with treatment outcomes. Future studies with larger participant sizes are needed to evaluate the role of SLPI in OPC risk and response to treatment stratified by HPV status.
